# Light storage for one second in room-temperature alkali vapor

**DOI:** 10.1038/s41467-018-04458-4

**Published:** 2018-05-30

**Authors:** Or Katz, Ofer Firstenberg

**Affiliations:** 1 Department of Physics of Complex Systems, Weizmann Institute of Science, Rehovot, 76100 Israel; 2 Rafael Ltd, IL-31021 Haifa, Israel

## Abstract

Light storage, the controlled and reversible mapping of photons onto long-lived states of matter, enables memory capability in optical quantum networks. Prominent storage media are warm alkali vapors due to their strong optical coupling and long-lived spin states. In a dense gas, the random atomic collisions dominate the lifetime of the spin coherence, limiting the storage time to a few milliseconds. Here we present and experimentally demonstrate a storage scheme that is insensitive to spin-exchange collisions, thus enabling long storage times at high atomic densities. This unique property is achieved by mapping the light field onto spin orientation within a decoherence-free subspace of spin states. We report on a record storage time of 1 s in room-temperature cesium vapor, a 100-fold improvement over existing storage schemes. Furthermore, our scheme lays the foundations for hour-long quantum memories using rare-gas nuclear spins.

## Abstract (web summary)

Storing quantum memories for a long time is important and challenging for quantum communication. Here the authors demonstrate a storage time of about 1 s using spin exchange relaxation free resonance in cesium vapor.

## Introduction

Atoms with incomplete electronic shells, most prominently alkali metals, have been central to the research on coherent light–matter interactions and their ensuing quantum technologies^1^. In particular, the first works on coherent light storage and quantum memories have utilized warm alkali vapors^2–5^, demonstrating the mapping of a photonic state to a long-lived collective state of the atomic ensemble. The realization of high-quality optical quantum memories is a key requirement for future optical quantum networks^6–9^.

The archetypal mechanism of light storage using alkali vapor is based on electromagnetically induced transparency (EIT), involving the signal field to be stored and a strong control field^2,10–12^. These fields resonantly couple one atomic excited state to two spin states within the ground level. Under EIT, there exists a long-lived dark state, which is the superposition of these spin states that is decoupled from the excited state due to destructive interference between the excitation pathways. While the control is on, the signal pulse entering the medium couples coherently to the dark state, forming a slowly-propagating polariton. Storage is done by turning off the control and stopping the polariton, thereby mapping the signal field onto a stationary field of dark-state coherence. Turning on the control retrieves the signal.

**Fig. 1:**
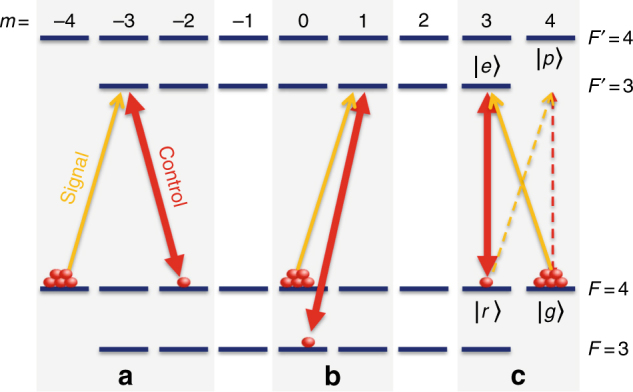
Configurations of light storage on the cesium ground-level. **a** Zeeman coherence $\left| {{\mathrm{\Delta }}m} \right|$ = 2. **b** Hyperfine coherence $\left| {\Delta m} \right|$ = 0. **c** Zeeman coherence $\left| {{\mathrm{\Delta }}m} \right|$ = 1, associated with spin orientation, used here. The dashed arrows represent an additional weak process, discussed in the text. In ^133^Cs, the ground and excited state hyperfine splittings are 9.19 and 1.17 GHz respectively, and the Doppler width of the optical linewidth is 370 MHz at *T* = 40 °C. Note that the signal is stored and retrieved as a linearly-polarized field, despite the fact that only one of its circular-polarization components (solid yellow line) enters the *Λ*-system $\left| g \right\rangle - \left| e \right\rangle - \left| r \right\rangle$, see Methods

In addition to the electron spin *S* = 1/2, alkali-metal atoms have a nuclear spin *I* > 0 (*I* = 7/2 for ^133^Cs) and thus possess multiple spin states. These are characterized by the hyperfine spin *F* = *I* ± *S* and its projection *m* on the quantization axis $\hat z$. Various combinations of spin states are accessible with different signal–control configurations, as shown in Fig. 1. Most light storage schemes utilize either the Zeeman coherence Δ*m* = 2 (Fig. 1a) or the hyperfine coherence Δ*m* = 0 (Fig. 1b)^13^. The relaxation of these coherences at high atomic densities is dominated by pairwise spin–exchange collisions^14^. During a collision, the valence electrons of the colliding pair overlap for a few picoseconds, accumulating a phase between the hybridized (singlet and triplet) electronic spins. While the total spin is conserved, the randomness of the collision parameters leads to relaxation of most ground-state coherences, limiting the storage lifetime in these schemes^2,13,15,16^.

It has been known, however, that the Zeeman coherence Δ*m* = 1, associated with the spin orientation moment, is unaffected by spin-exchange collisions at low magnetic fields^17–19^. This property is the underlying principle of spin-exchange relaxation-free (SERF) magnetometers, currently the most sensitive magnetic sensors^20,21^. Here, by mapping the signal onto the Δ*m* = 1 coherence (Fig. 1c), we realize light storage that benefits from the SERF mechanism at low magnetic field and report on a record storage time of up to 1 s in cesium vapor.

## Results

### Storage scheme and lifetime

**Fig. 2:**
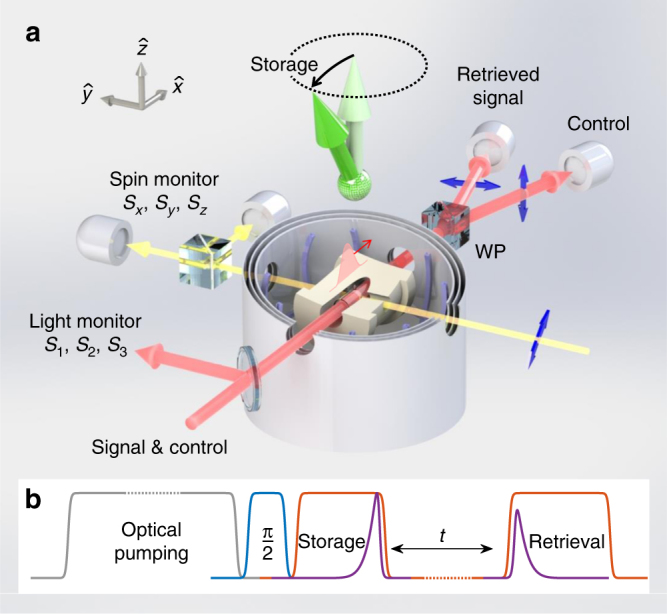
Experimental setup and sequence. A cylindrical vapor cell with diameter 10 mm and length 30 mm is held inside a hot-air oven (**a**, beige), enclosed by three Helmholtz coil pairs and magnetic shield layers. First, optical pumping (**b**, gray) is done along $\hat x_{}^{}$ using two circularly-polarized beams (not shown in **a**) for the two hyperfine sublevels. Subsequently, a *π*/2 pulse of magnetic field along $\hat y$ (**b**, blue) prepares a spin ensemble oriented along $\hat z$ (**a**, light green arrow). The control and incoming signal (**a**, red beam) are sampled before the cell to monitor their polarization state. Storage and retrieval of a signal pulse (**b**, purple) is done by turning off and then on the control (**b**, red). The light state is stored onto the orientation of the tilted spin polarization (**a**, dark green arrow), which can be monitored with auxiliary far-detuned light (**a**, yellow beam). The retrieved signal is separated from the control using a high extinction-ratio Wollaston prism (WP). Blue arrows in **a** indicate optical polarizations

A paraffin-coated vapor cell is at the heart of the experimental system, shown in Fig. 2a. We zero the magnetic field to better than $\left| {{B}} \right|$ < 1 μG and control the cesium density *n*(*T*) via the cell temperature *T*. The experimental sequence is shown in Fig. 2b. We initially orient the atoms along the optical axis $\hat x$ using optical pumping (polarization >95%). We then rotate the polarized spins onto our quantization axis $\hat z$ using a short pulse of magnetic field along $\hat y$, thus preparing them in the state $\left| g \right\rangle$ ≡ $\left| {F = 4,m = 4} \right\rangle$. Subsequently, we turn on the control field and a small magnetic field *B*_*z*_ ≤ 15 μG. The control field is linearly polarized along $\hat z$ and resonant with the $\left| r \right\rangle = \left| {F = 4,m = 3} \right\rangle$ → $\left| e \right\rangle = \left| {F\prime = 3,m\prime = 3} \right\rangle$ transition.

**Fig. 3:**
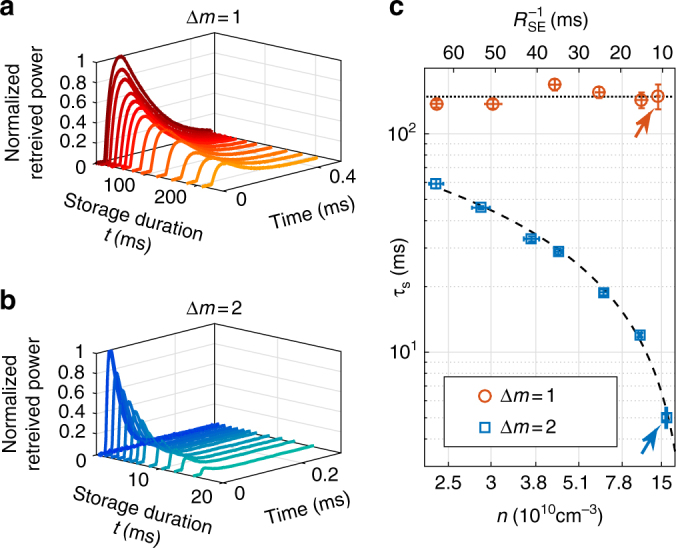
Storage lifetime. **a**, **b** Retrieved pulses for several storage durations in our scheme Δ*m* = 1 (**a**), compared to the standard scheme Δ*m* = 2 (**b**). The lifetime in our scheme is longer than 100 ms, compared to only a few ms in the standard scheme. **c** Storage lifetime as a function of atomic density *n* and spin-exchange rate *R*_SE_ in our scheme (red), which is unaffected by collisions and thus remains constant (dotted line). In contrast, the lifetime in the standard scheme (blue) is well described by a linear fit (dashed line). Data in **a**, **b** correspond to points marked by arrows in **c**. The two schemes exhibit comparable storage lifetimes only at low atomic densities, where the optical depth and thus the storage efficiency are compromised^3^. Vertical error bars are 95%-confidence intervals of the exponential fit to storage efficiency; horizontal error bars are 95%-confidence intervals of the spin exchange rate, measured at high magnetic fields before and after each experimental sequence

With the control on, we send a weak signal pulse, linearly polarized along $\hat y$. The signal couples to the $\left| g \right\rangle - \left| r \right\rangle$ coherence, orienting the spins while propagating (other coupled transitions and additional experimental details are discussed in Methods). We store the signal field onto spin orientation by turning off the control and, after a duration *t*, retrieve it by turning the control back on. As a reference, we perform light storage in a standard Δ*m* = 2 scheme with circularly-polarized signal and control fields^2^. At a density of *n* = 1.4 × 10^11^ cm^−3^ (*T* ≈ 40 °C), the two schemes exhibit comparable (internal) storage efficiency, 9% for the Δ*m* = 2 scheme and 14% for the Δ*m* = 1 scheme, with no particular optimization of the temporal shape of the control and signal^16^. Figure 3a, b shows the retrieved pulses for both schemes. We extract the storage lifetime *τ*_s_ by fitting the retrieved power to the decay function exp (−*t*/*τ*_s_). Light storage on spin orientation exhibits a remarkable lifetime *τ*_s_ = 149(20) ms in this experiment, much longer than the 5.0(3) ms obtained with the standard scheme.

To study the effect of spin-exchange collisions, we tune the collision rate *R*_SE_ = *αn*(*T*) by changing *T* (*α* = 6.5 × 10^−10^ cm^3^ s^−1^ near room temperature)^22^. Figure 3c shows the measured storage lifetime versus *R*_SE_. The relaxation of the Δ*m* = 2 coherence is dominated by spin exchange, as indicated by the linear dependence of *τ*_s_ on *R*_SE_ in the standard scheme. In contrast, our scheme is found to be insensitive to *R*_SE_, affirming that the Δ*m* = 1 coherence is conserved under spin exchange. We conclude that storage on spin orientation maintains long memory lifetimes at elevated optical depths. The observed lifetime *τ*_s_ = 150 ms is limited by the spin-destruction time at low magnetic fields, measured *T*_1_ = 300 ± 100 ms in our system.

### Coherence of the storage scheme

**Fig. 4:**
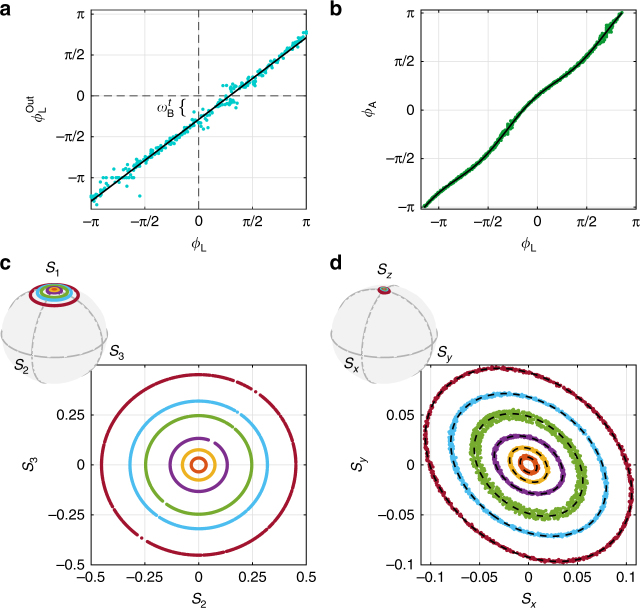
Mapping from light polarization to spin orientation and back. **a** The measured phase of the retrieved light after storage for *t* = 100 ms follows the input phase up to an offset (line is a linear fit with unity slope). **b** The azimuthal angle of the collective atomic spin versus the incoming optical phase (line is a fit to elliptical mapping, see Supplementary Note 1). **c** Light polarization visualized on the Poincaré sphere and projected onto the transverse plane. **d** Collective atomic spin visualized on the Bloch sphere and projected onto the transverse plane. Data in **b**–**d** taken 1 ms after storage (without retrieval)

We confirm the coherent nature of our storage scheme by measuring for *t* = 100 ms the phases of the input signal *ϕ*_L_ and output signal $\phi _{\mathrm{L}}^{{\mathrm{out}}}$, as shown in Fig. 4a. Larmor precession during storage leads to a constant offset between $\phi _{\mathrm{L}}^{{\mathrm{out}}}$ and *ϕ*_L_. The Larmor frequency in this experiment was measured independently to be *ω*_*B*_ = 1.34(6) × 2*π* Hz, predicting a rotation of *ω*_B_*t* = 0.84(4), in agreement with the observed offset $\phi _{\mathrm{L}} - \phi _{\mathrm{L}}^{{\mathrm{out}}} = 0.9(2)$.

To explain the immunity to spin-exchange collisions, we first explore the light-atom mapping. Taking the control field as a phase reference, the signal properties, or the light state to be stored, are encompassed in the polarization of the incoming (signal + control) field. This polarization is visualized on the Poincaré sphere using the Stokes parameters *S*_1_, *S*_2_, *S*_3_ in Fig. 4c. To characterize their mapping onto the atomic spins, we monitor the spins during storage using polarization rotation of a far-detuned beam. The spin state of the ensemble is described by the collective electronic spin $\vec s$ = (*s*_*x*_, *s*_*y*_, *s*_*z*_), defined by $\vec s = \frac{1}{N}\mathop {\sum}\nolimits_i \left\langle {\vec s^i} \right\rangle$, where $\vec s^i$ is the spin operator of the *i*th atom and *N* the number of atoms^23^. These are visualized on the Bloch sphere in Fig. 4d.

From the Stokes operators, we extract for the light the complex ratio $i\eta _{\mathrm{L}}e^{i\phi _{\mathrm{L}}} = E_{\mathrm{s}}{\mathrm{/}}E_{\mathrm{c}}$ between the signal amplitude *E*_s_ and the control amplitude *E*_c_. The phase *ϕ*_L_ is constant in space and time during an experimental sequence. As the signal is much weaker than the control $\eta _{\mathrm{L}} \ll 1$, the Stokes vector is located near the north pole of the Poincaré sphere (Fig. 4c). Initially, with only the control on (*η*_L_ = 0), the atomic spins are oriented along the $\hat z$ direction, corresponding to the north pole of the Bloch sphere. This initialized spin orientation underlies the difference between our system and those previously demonstrated with the same fields configuration^10,13,24^. The incoming signal tilts the collective spin off the pole, producing a transverse spin component (Fig. 4d).

The light state has a polar angle *η*_L_ and an azimuth *ϕ*_L_; the corresponding atomic state has a polar angle *η*_A_ = $\sqrt {s_x^2 + s_y^2} {\mathrm{/}}s_z$ and azimuth *ϕ*_A_ = arctan(*s*_*y*_/*s*_*x*_). We find that the storage procedure maps the light quadratures *S*_2_, *S*_3_ onto the spin components *s*_*y*_, *s*_*x*_ by transforming circles into nearly-circular ellipses with *ϕ*_A_ ≈ *ϕ*_L_, see Fig. 4b. We conclude that the light state is mapped onto the atomic spin orientation.

### Storage of light for 1 s

**Fig. 5:**
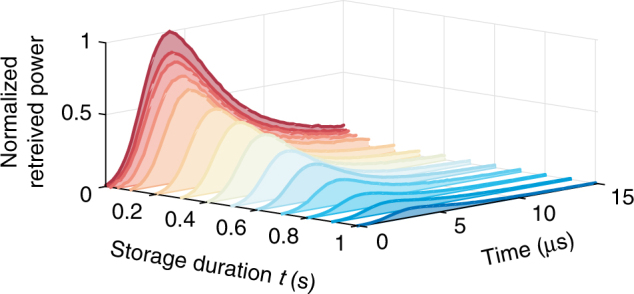
1 s long light storage. Light storage for up to *t* = 1 s of short (*τ*_p_ = 5.5 μs) signal pulses

Upon completion of the measurements reported above, we kept the vapor cell warm at *T* = 45 °C for a week, keeping the stem cold at *T* = 25 °C, and performed storage experiments for up to *t* = 1 s at room temperature. As shown in Fig. 5, we observed a 1/*e* storage time of *τ*_s_ = 430(50) ms, indicating that the temperature cycle lowered the spin destruction, presumably due to curing of the coating^25^. In conjunction with the signal pulse duration of *τ*_p_ = 5.5 μs, we thus obtained an extremely large fractional delay of *τ*_s_/*τ*_p_ ≈ 80,000.

## Discussion

The immunity to spin-exchange collisions can easily be understood in the absence of hyperfine interaction (for example if *I* = 0), as the total spin, and hence the orientation of the electronic spin, is conserved under these collisions. However when *I* > 0, it is not trivial to see how also the entanglement between the electronic and nuclear spins is conserved. To understand the case *I* > 0, we examine a collision between two cesium atoms. After a weak classical signal is stored, the state of the *i*th atom is $\left| {\psi _i} \right\rangle$ = $\left| {g_i} \right\rangle + \sqrt 2 \eta _{\mathrm{A}}e^{i\phi _{\mathrm{A}}}\left| {r_i} \right\rangle$, with $\eta _{\mathrm{A}} \ll 1$. Collision are brief relatively to the hyperfine frequency and thus affect only the electronic spins. We therefore decompose the stored state into the electronic $\left| { \uparrow _i} \right\rangle \equiv \left| {s_z^i = \frac{1}{2}} \right\rangle$, $\left| { \downarrow _i} \right\rangle \equiv \left| {s_z^i = - \frac{1}{2}} \right\rangle$ and nuclear $\left| { \Uparrow _i} \right\rangle \equiv \left| {I_z^i = I} \right\rangle$, $\left| { \Downarrow _i} \right\rangle \equiv \left| {I_z^i = I - 1} \right\rangle$ spin components, writing $\left| {\psi _i} \right\rangle = \left| { \uparrow _i \Uparrow _i} \right\rangle$ + $\sqrt 2 \eta _{\mathrm{A}}e^{i\phi _{\mathrm{A}}}\left( {q\left| { \uparrow _i \Downarrow _i} \right\rangle + p\left| { \downarrow _i \Uparrow _i} \right\rangle } \right)$. For cesium, $I = \frac{7}{2}$ and *p*^2^ = 1 − *q*^2^ = $\frac{1}{8}$ (note that the following arguments are general and independent of these values). The exchange interaction during a collision between a pair of atoms introduces a random phase *χ* between their hybrid electronic states—the singlet and triplet^14^. The pair, initially in the product state $\left| {\psi _{ij}} \right\rangle = \left| {\psi _i} \right\rangle \left| {\psi _j} \right\rangle$, leaves the collision in the state $\left( {P_T^{i,j} + e^{i\chi }P_S^{i,j}} \right)\left| {\psi _{ij}} \right\rangle$, where $P_S^{i,j}$ = $\left( {\left| { \uparrow _i \downarrow _j} \right\rangle - \left| { \downarrow _i \uparrow _j} \right\rangle } \right)$ $\left( {\left\langle { \uparrow _i \downarrow _j} \right| - \left\langle { \downarrow _i \uparrow _j} \right|} \right)$/2 and $P_T^{i,j} = 1 - P_S^{i,j}$ are the singlet and triplet projection operators. Yet for weak signals, at the limit *η*_A_ → 0, the colliding pair is a nearly-perfect spin triplet, possessing a negligible singlet component $\left\langle {\psi _{ij}} \right|P_S^{i,j}\left| {\psi _{ij}} \right\rangle$ = $4(pq)^2\eta _{\mathrm{A}}^4 \to 0$. Therefore, the random phase *χ* is inconsequential, and the pair state is immune to spin-exchange relaxation.

It is also instructive to examine the quantum limit, where the signal has at most a single photon in the state $\alpha \left| 0 \right\rangle + \beta \left| 1 \right\rangle$, where *α* and *β* are the SU(2) parameters of a qubit with either zero $\left| 0 \right\rangle$ or one $\left| 1 \right\rangle$ photons. At storage, the initial collective atomic state $\left| G \right\rangle = \mathop {\prod}\nolimits_i \left| {g_i} \right\rangle$ is transformed into $\left| R \right\rangle = (\alpha + \beta F_ - )\left| G \right\rangle$, where *F*_−_ = $\frac{1}{\sqrt {N}}\mathop {\sum}\nolimits_i \left( {s_ - ^i + i_ - ^i} \right)$ is the collective spin operator accounting for the Δ*m* = 1 transition. One can verify that $\left| R \right\rangle$ is an exact triplet for any atom pair (*i*, *j*), since $\left| G \right\rangle$ is a triplet and $\left[ {P_T^{i,j},F_ - } \right] = 0$. Therefore, the stored qubit $\left| R \right\rangle$ is fully conserved under spin exchange.

The analyses above are valid for a highly-polarized ensemble, where atoms mostly populate the end state $\left| g \right\rangle$. This regime has been identified by Jau et al.^26^, who demonstrated the suppression of spin-exchange spectral broadening for the so-called end resonance. However, the ensemble remains highly-polarized only at short times after storage $t \ll T_1$, whereas at later times *t* ≳ *T*_1_, the spin state follows a spin-temperature distribution along $\hat z$^27^. While the degree of polarization decreases, most spin moments decay by spin-exchange collisions, except for the orientation moment (Δ*m* = 1 coherence), which is conserved by the aforementioned SERF mechanism^18^ when the magnetic field is sufficiently low [ideally $\omega _{\mathrm{B}}$ ≲ $\left( {T_1T_{{\mathrm{SE}}}} \right)^{ - 1/2}$ ≪ $T_{{\mathrm{SE}}}^{ - 1}$]. The SERF mechanism, utilized in precision magnetometers^21^, has been extensively characterized under the conditions of constant polarization^28^. In our storage experiment, the polarization decreases continuously due to *T*_1_ relaxation, while still maintaining a spin-temperature distribution and satisfying the SERF conditions. It is therefore the combination of the end-resonance^26^ and SERF^18^ mechanisms that prevents the decoherence due to spin exchange in our storage scheme.

We note that a mapping $\eta _{\mathrm{L}}e^{i\phi _{\mathrm{L}}} \leftrightarrow \eta _{\mathrm{A}}e^{i\phi _{\mathrm{A}}}$ with nonzero ellipticity is non-ideal for quantum memories, as it links the retrieval amplitude to the (azimuthal) phase and thus distorts the quantum state. In our experiment, the ellipticity originates from polarization self-rotation^29^ due to the off-resonance Raman process $\left| g \right\rangle - \left| p \right\rangle - \left| r \right\rangle$ (dashed arrows in Fig. 1c) weakly perturbing the ideal EIT process $\left| g \right\rangle - \left| e \right\rangle - \left| r \right\rangle$. When the strength of these processes is comparable, the resulting so-called Faraday interaction limits the storage to only one quadrature, compressing the ellipse into a line^23^. In our scheme, *Δ* > *Γ*, where *Δ* = 1.17 GHz is the detuning from the $\left| g \right\rangle - \left| p \right\rangle$ resonance, and *Γ* = 185 MHz is the Doppler half-linewidth, so the dark state qualitatively obtains the form $\left| g \right\rangle$ + $\sqrt 2 \eta _{\mathrm{L}}\left( {e^{i\phi _{\mathrm{L}}} - \epsilon e^{ - i\phi _{\mathrm{L}}}} \right)\left| r \right\rangle$, with $\epsilon$ = (1 − *iΔ*/*Γ*)^−1^; in cesium, *Δ* = 6.5*Γ*, yielding ellipticity of order 0.15. An ideal mapping $\epsilon$ → 0 with ${{\Delta }} \gg {{\Gamma }}$ can be approached, for example, by storing on the lower hyperfine ground-level $\left( {\left| r \right\rangle \Rightarrow \left| {F = 3,m = 3} \right\rangle } \right)$, which benefits from a larger detuning from other transitions (*Δ*/*Γ* = 45) and still maintains the spin-exchange resistance. We derive in the Supplementary Note 3 the exact analytical form of the mapping $\eta _{\mathrm{L}}e^{i\phi _{\mathrm{L}}} \leftrightarrow \eta _{\mathrm{A}}e^{i\phi _{\mathrm{A}}}$ and further develop a procedure employing a magnetic field *B*_*z*_ that corrects for and eliminates the ellipticity. It follows that the ellipticity is non-fundamental and amendable.

In conclusion, our light-storage scheme demonstrates record lifetimes of hundreds of milliseconds at room temperature. Currently we employ polarization filtering to remove the control light from the retrieved signal. Quantum memories relying only on polarization filtering are adequate for storing weak coherent states^30^ and squeezed states^31^. Utilizing the lower hyperfine manifold, as outlined above, would additionally allow for frequency filtering, adequate for memory applications at the single photon level^32^. In addition, our scheme paves the way towards the coupling of photons to ultra-stable nuclear spins. Rare isotopes of noble gases, such as helium-3, carry nuclear spins that are optically inaccessible and exhibit coherence times on the scale of hours^33^. It has been demonstrated that spin-exchange collisions can coherently couple the orientation moment of two alkali species^34,35^ as well as the orientation moment of alkali and rare-gas atoms^36^, while all other spin moments are relaxed. Mapping light onto spin orientation thus not only protects the stored information from self spin-exchange relaxation, but also enables its transfer from one spin ensemble to another. Therefore, combined with coherent spin-exchange interaction between alkali and rare gases, our scheme is potentially a key element in employing and manipulating nuclear spins for quantum information applications.

## Methods

### Additional experimental details

The ensemble is initially prepared via optical pumping using two auxiliary, circularly-polarized laser beams, which cover the entire cell area. The beams are derived from two DBR diode-lasers at 894 nm, tuned to resonate with the *D*_1_ optical transition: one beam (20 mW, tuned to the *F*_*g*_ = 3 → *F*_*e*_ = 4 transition) pumps the atoms out of the lower hyperfine manifold, and the other other beam (4 mW, tuned to the *F*_*g*_ = 4 → *F*_*e*_ = 4 transition) pumps the atoms within the upper hyperfine manifold to the end state $\left| g \right\rangle$. The windows in the system (oven and cell) are made of non-birefringent glass, and the circular polarization of the pumping beams is verified with a polarization analyzer both before and after the cell. The signal and control fields originate from a single DBR diode-laser coupled into a single-mode fiber, followed by high-quality film linear polarizer and a closed-loop intensity-noise eater. These provide for a beam in a single Gaussian mode, linearly polarized, and amplitude stabilized. The signal is split from the control using a polarizing beam splitter (PBS). Each beam is passed through an acusto-optic modulator, driven by a frequency-locked RF generator, which provide for independent control of the fields’ amplitude and precise control of their frequency and relative phase during the experimental sequence. The beams are recombined using a second PBS and expanded to cover the entire cell area. A Soleil-Babinet compensator (SBC) immediately before the cell precisely corrects for any residual polarization rotation occurred in the system (for example on the mirrors). A second SBC after the cell and a high-extinction-ratio Wollaston prism filter out the control light to a level better then 10^−5^, corresponding to roughly a million noise photons per pulse. The second SBC is tuned prior to the experiment by sending far-detuned control light into an unpolarized vapor and minimizing the light at the signal readout channel. For the Δ*m* = 2 scheme (the control experiment), additional quarter-wave plates are introduced before and after the cell. The paraffin coated cell is a cylinder, with an inner diameter of 9 mm and a length of 30 mm. A cesium droplet is kept cooler than the cell walls at a predefined cold finger. The cell exhibits a lifetime of *T*_1_ = 300 ± 100 ms at low magnetic fields and a lifetime of 1 s at higher fields (before our curing procedure). Lifetimes and residual fields were measured by optical pumping followed by free precession and decay in the dark, monitored by Faraday rotation of a probe beam. The probe was tuned to the middle of the *D*_1_ transitions, being sensitive to the polarization of both ground-state hyperfine manifolds. Our *T*_2_ times were spin-exchange limited at high magnetic fields, and approached the *T*_1_ lifetime at low magnetic fields when operated in SERF mode. We observed a (not-permanent) decrease in the *T*_1_ lifetime when operating above 50 °C.

### Experimental calibration

We perform calibration experiments prior to storage, adjusting the setup parameters to produce no output signal in the absence of an incoming signal. In particular, we fine tune the spin rotation from $\hat x$ to $\hat z$ after optical pumping and zero the transverse magnetic fields (*B*_*x*_ and *B*_*y*_). This calibration is important, as light storage based on $\left| {{\mathrm{\Delta }}m} \right|$ = 1 coherence is particularly sensitive to experimental imperfections affecting the collective spin orientation: Nonzero transverse magnetic fields (*B*_*x*_, *B*_*y*_ ≠ 0) tilt the collective spin during storage and produce a small transverse spin component, which is subsequently mapped to an output signal even without an input signal. Additionally, misalignment between the direction of the (linear) polarization of the control field and the initial polarization direction of the spin ensemble manifests as a nonzero transverse spin (when identifying the control polarization as the quantization axis), again producing an output signal for no input signal. We thus properly align the initial spin polarization direction and validate that *B*_*x*_ and *B*_*y*_ are truly zeroed by verifying the absence of output signal for all “storage” durations *t*, confirming that there is no tilt of the spin during the experiment.

### Phase uniformity

In the storage experiments, we send weak signal pulses of durations *τ*_p_ = 0.03−0.15 ms, linearly polarized along $\hat y$, and having the same spatial mode and frequency as the control field. The corresponding range of pulse bandwidths $2\tau _{\mathrm{p}}^{ - 1} \approx 2 - 10 \times 2\pi$ kHz is comparable to the width of the EIT transmission window and much larger than the Larmor precession rate *ω*_B_, such that the relative phase between the signal and control fields is constant during a storage experiment. The signal and control beams cover the entire cell area, with their wave-vector difference much smaller than the inverse cell width. This was chosen, because the storage lifetime is sensitive to the spatial-mode overlap of the signal and control fields. Specifically, an angular deviation between them yields a spatial phase grating, which is imprinted on the collective spin wave. Dephasing of this spin wave due to thermal atomic motion limits the storage lifetime^37^, as it does regardless of the exact spin coherence used. However for the $\left| {{\mathrm{\Delta }}m} \right|$ = 1 scheme, the spin-wave grating manifests as a spatially varying orientation, impairing the resistance to spin-exchange collisions. Colliding spins with different orientations are no longer perfect triplets (the singlet component $\left\langle {\psi _{ij}} \right|P_S^{i,j}\left| {\psi _{ij}} \right\rangle$ grows quadratically with the angle between the colliding spins), which can be explained by their reduced indistinguishability due to the spatially-varying mapping. Consequently, the $\left| {{\mathrm{\Delta }}m} \right|$ = 1 scheme loses its spin-exchange immunity if the signal and control beams are misaligned. Prior to the experiment, we align the signal and control beams such that their relative phase is spatially uniform. This is achieved by sampling them before the cell and monitoring their spatial interference pattern using a camera at two different positions. A rotated polarizer allows for their interference. In both positions, we verify that no interference fringes appear, obtaining spatial uniformity of the relative-phase to better than 10%.

### Measuring light and spin polarizations

To measure the Stokes parameters of the light and extract both *η*_L_ and *ϕ*_L_, we sample the optical fields before the cell. To measure the collective atomic spin, we use a weak monitor beam propagating along $\hat y$, linearly polarized ($\hat z$) and red-detuned 22 GHz from the $\left| g \right\rangle \to \left| e \right\rangle$ transition. Far from resonance, the polarized atoms render the medium optically chiral, rotating the polarization of the monitor beam in the *xz* plane by an angle *θ* = *βs*_*y*_ via the linear Faraday interaction, where *β* is a constant^22^. We measure *θ* after the cell using a balanced detector^35^. The collective spin during storage is measured by increasing the magnetic field to *B*_*z*_ = 4 mG, making the spin precess around the $\hat z$ axis at a frequency *ω*_B_ = 1.4 · 2*π* kHz and thus modulating *θ* in time according to *θ* = *C* cos(*ω*_B_*t* + *ϕ*_A_). We identify the transverse spin components at storage as *s*_*x*_ = *C* cos(*ϕ*_A_)/*β* and *s*_*y*_ = *C* sin(*ϕ*_A_)/*β*. We scan the input phase *ϕ*_L_ at various signal powers and measure *s*_*x*_, *s*_*y*_ in each realization. We perform an additional set of experiments by applying *B*_*x*_ instead of *B*_*z*_, thus modulating the spin in the *yz* plane and measuring $\overline {s_z}$ averaged per signal power. With *s*_*x*_, *s*_*y*_, and $\overline {s_z}$, we extract $\eta _{\mathrm{A}} = \sqrt {s_x^2 + s_y^2} {\mathrm{/}}\overline {s_z}$ and *ϕ*_A_ = arctan(*s*_*y*_/*s*_*x*_). For each measurement, we use the normalized vector $\vec s{\mathrm{/}}\left\| {\vec s} \right\|$ to lay the spin on the Bloch sphere and eliminate *β*, which is independent of the signal parameters^22^.

### Completeness of the optical transition

It is instructive to discuss an intricacy that arise when the quantization axis ($\hat z$) is orthogonal to the light propagation axis ($\hat x$) in the special case of an ensemble initially polarized (oriented) along $\hat z$. With a control field linearly-polarized along $\hat z$, the maximally-polarized atoms are confined to a single $\left| {{\mathrm{\Delta }}m} \right| = 1$ transition. From the viewpoint of the quantization axis $\hat z$, this transition corresponds to the *Λ*-system $\left| g \right\rangle - \left| e \right\rangle - \left| r \right\rangle$, as depicted in Fig. 1. Importantly, the signal mode is completely stored and retrieved via this transition, despite the fact that its linear polarization ($\hat y$) decomposes to two circular polarizations, of which only one is included in the $\left| g \right\rangle - \left| e \right\rangle - \left| r \right\rangle$ system. Under the reduced Maxwell equations, including the transverse susceptibility tensor of the medium^22^, the normal modes comprises only *E*_*y*_ and *E*_*z*_ components (recall that the non-evanescent field polarization remains within the transverse plane during praxial propagation). Consequently, the signal mode *E*_*y*_ is stored and retrieved as a whole. The optical depth for the signal is however reduced by the Clebsch-Gordan coefficient of the $\left| g \right\rangle - \left| e \right\rangle$ transition.

### Data availability

The data that support the findings of this study are available from the corresponding author on reasonable request.

## Electronic supplementary material

Supplementary Information
