# Supplementary material for: Light storage for one second in room-temperature alkali vapor
Source: Nat Commun. 2018 May 30;9:2074. doi: 10.1038/s41467-018-04458-4 (PMC5976718; doi:10.1038/s41467-018-04458-4)
Supplement: Supplementary file 1 — Supplementary Information [file 41467_2018_4458_MOESM1_ESM.pdf]

# Supplementary Information

for

# Light storage for one second in room-temperature alkali vapor

Or Katz and Ofer Firstenberg.

In this Supplementary Information, we analytically derive the mapping from light to atoms $\eta_{\rm L} e^{i\phi_{\rm L}} \rightarrow \eta_{\rm A} e^{i\phi_{\rm A}}$ (supplementary note 1) and from atoms to light $\eta_{\rm A} e^{i\phi_{\rm A}} \rightarrow \eta_{\rm L}^{\rm out} e^{i\phi_{\rm L}^{\rm out}}$ (supplementary note 2). Subsequently, we examine the overall mapping between the input and output signals and present a method to eliminate its ellipticity (supplementary note 3).

## Supplementary note 1. Storage: mapping light to atoms

In light storage based on EIT, the atomic state during storage corresponds to the EIT dark state. Here we derive the dark state by analyzing the non-Hermitian Hamiltonian of the system. Non-Hermitian Hamiltonian dynamics with stochastic quantum jumps is used to solve open-systems dynamics [1]. In EIT, population of the excited state is avoided, thus the effect of the quantum jumps in determining the dark state is small. For our $\Delta m = 1$ scheme, the control field $E_{\rm c}$ and signal field $E_{\rm s}$ comprise the total electric field as

$$\mathbf{E} = E_{\rm c}\hat{\mathbf{z}} + E_{\rm s}\hat{\mathbf{y}} = E_c\left(\hat{\mathbf{z}} + i\eta_{\rm L} e^{i\phi_{\rm L}}\hat{\mathbf{y}}\right). \tag{1}$$

In principle, the ground level of the Cs atoms have many spin states that are coupled by the signal and control fields. However, we use optical pumping to initialize the atoms in the maximally polarized state $|g\rangle$ (see Fig. 1 in the main text), while the weak signal field with $\eta_{\rm L} \ll 1$ varies this state only perturbatively. We therefore consider here, in addition to $|g\rangle$, only the states $|r\rangle$,$|e\rangle$,$|p\rangle$, which couple to $|g\rangle$ to first order in $\eta_{\rm L}$. The non-Hermitian Hamiltonian is given by $H = H_0 + V$, where

$$\begin{aligned} H_0 &= -i\Gamma\,|e\rangle\langle e| + (\Delta - i\Gamma')\,|p\rangle\langle p| \\ V &= \Omega_{\rm s}\,|g\rangle\langle e| + \Omega_{\rm c}\,|r\rangle\langle e| + a_{\rm Cs}\Omega_{\rm c}\,|g\rangle\langle p| + b_{\rm Cs}\Omega_{\rm s}\,|r\rangle\langle p| + \text{h.c.} \end{aligned} \tag{2}$$

Here $\Delta = 1100\times 2\pi$ MHz is the excited-level hyperfine splitting, and we take $\Gamma = 184\times 2\pi$ MHz, $\Gamma' = 124\times 2\pi$ MHz for the half linewidth of the Doppler-broadened optical transition, at $T = 40\mathrm{C}^\circ$. The Rabi frequencies are given by $\Omega_{\rm c} = d_{\rm Cs}\,|E_{\rm c}|\,/(\sqrt{2}\hbar)$ and $\Omega_{\rm s} = \sqrt{2}\Omega_c\eta_{\rm L} e^{i\phi_L}$, where $d_{\rm Cs} = 2.6\cdot\frac{\sqrt{7}}{4}ea_0$ is the dipole moment transition element for the Cs D1 transition, with the electron charge $e$ and Bohr radius $a_0$ [2]. The ratios of the Clebsch-Gordan coefficients between the two $\Lambda$ systems is $a_{\rm Cs} = 4/\sqrt{7}$ and $b_{\rm Cs} = 1/\sqrt{7}$.

Because of the off-resonant coupling to the state $|p\rangle$, the system has no dark state, as manifested by the fact that $H$ has no zero eigenvalues. However, one can identify a quasi-dark state — the eigenstate of $H$ with the lowest imaginary eigenvalue. For our Hamiltonian, the lowest imaginary eigenvalue $\lambda_{\rm min} \propto -i\Gamma\Omega_{\rm c}^2/\Delta^2$ accounts for the loss of population from the dark state to other ground-level states via off-resonant pumping. Diagonalizing $H$, we find the corresponding eigenstate to first order in $\eta_{\rm L}$ and in $\Gamma/\Delta$,

$$|\psi_{\rm A}\rangle = |g\rangle + \sqrt{2}\eta_{\rm L} e^{-3i\alpha}[e^{i(\phi_{\rm L}-\alpha)} \underbrace{-i\alpha e^{-i(\phi_{\rm L}-\alpha)}}_{\text{perturbation to the ideal dark state}}]\,|r\rangle \tag{3}$$

with $\alpha = f_{\rm Cs}\Gamma/\Delta$, and $f_{\rm Cs} = 0.58$ is a constant derived from the Clebsch-Gordan coefficients. To relate this state to the Bloch sphere representation, we write it as

$$|\psi_{\rm A}\rangle = |g\rangle + \sqrt{2}\eta_{\rm A} e^{i\phi_{\rm A}}\,|r\rangle \tag{4}$$

and identify the Bloch spin quadratures

$$s_x = \frac{1}{2}\eta_{\rm A}\cos(\phi_{\rm A})\,;\quad s_y = \frac{1}{2}\eta_{\rm A}\sin(\phi_{\rm A})\,;\quad s_z = \frac{1}{2}.$$

The parameters $\eta_{\rm A}$ and $\phi_{\rm A}$ thus serve as the angles on the Bloch sphere. With $\alpha \neq 0$, the state (3) manifests a non-ideal mapping from the Poincaré sphere to the Bloch sphere. From supplementary equations (4) and (3), we find the $\eta_{\rm L} e^{i\phi_{\rm L}} \rightarrow \eta_{\rm A} e^{i\phi_{\rm A}}$ mapping

$$\eta_{\rm A} = \eta_{\rm L}\sqrt{1 - 2\alpha\sin(2(\phi_{\rm L} - \alpha))} \tag{5}$$

and

$$\phi_\mathrm{A} = -\frac{\pi}{4} - 3\alpha + \arctan\left[\frac{1-\alpha}{1+\alpha}\tan\left(\phi_\mathrm{L} + \frac{\pi}{4} - \alpha\right)\right]. \tag{6}$$

These are the mathematical equations of an ellipse with the semi-major and semi-minor axes $\eta_\mathrm{L}(1 \pm \alpha)$ rotated by an angle $\left(\frac{\pi}{4} - \alpha\right)$. Mathematically, the phase $\phi_\mathrm{L}$ serves as the eccentric anomaly of the ellipse in the Bloch sphere. The mapping is exemplified graphically in supplementary figure 1 for $\eta_\mathrm{L} = 10^{-3}$ and $\alpha = 0.1$ with $0 \leq \phi_\mathrm{L} \leq 2\pi$. The ellipticity of the mapping, determined by the parameter $\alpha$, is manifested by a dependence of the tilt angle of the spin on the azimuthal phase.

As expected, in the ideal case $\alpha \to 0$ (for $\Gamma \ll \Delta$), the quasi-dark state in supplementary equation (3) becomes the ideal dark state, and supplementary equations . (5) and (6) reduce to the simple linear relations $\eta_\mathrm{A} = \eta_\mathrm{L}$ and $\phi_\mathrm{A} = \phi_\mathrm{L}$.

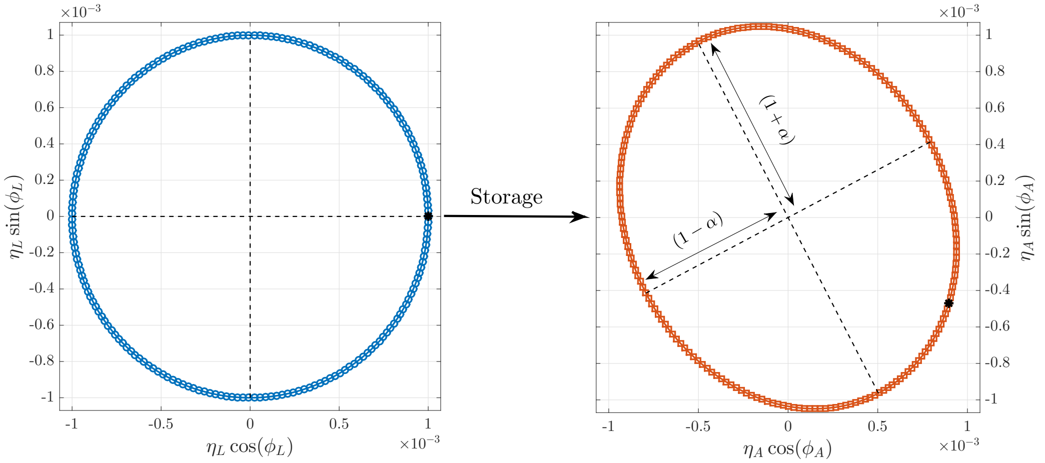


Supplementary figure 1: **Mapping of the light state to atomic spin state at storage.** The tranformation of the photonic input state represented on the Poincare sphere to the atomic state represented on the Bloch sphere is shown for $\eta_\mathrm{L} = 10^{-3}$, $\alpha = 0.1$. We mark the points $\phi_\mathrm{L} = 0$ and $\phi_\mathrm{A}(\phi_\mathrm{L} = 0)$ (black asterisks) to illustrate the transformation of the azimuthal phase, which acquires a mean additional phase shift of $-3\alpha$.

## Supplementary note 2. Retrieval: mapping atoms to light

The retrieval process could be described as the reverse process of storage [3, 4]. Nevertheless, it is instructive to consider a complementary formalism, which we give in this supplementary note. During the retrieval of the signal, similarly to storage, the atomic excitation changes adiabatically the incoming light field (control only) to a new light field (control+signal) that minimizes the loss. A formalism for describing the propagation of the electric field through the medium was introduced by Happer *et al.* [5]. They write the propagation equation of the field amplitude $\vec{E}$ as

$$\frac{d}{d\tilde{x}}\mathbf{E} = 2\pi i k n \left\langle \overleftrightarrow{\chi} \right\rangle_\perp \mathbf{E}, \tag{7}$$

where $k$ is the wave-number of the laser, $n$ is the atomic density, and $\tilde{x} = x - ct$ is the transformed spatial coordinate of the pulse. This equation relates the electric field vector in the medium $\mathbf{E} = (E_y, E_z)$ to the atomic state $|\psi_\mathrm{A}\rangle$ using the mean transverse susceptibility tensor of the atoms $\left\langle \overleftrightarrow{\chi} \right\rangle_\perp = \left\langle \psi_\mathrm{A} \middle| \overleftrightarrow{\chi} \middle| \psi_\mathrm{A} \right\rangle_\perp$, where $\chi_{ij} = d_i d_j / (\Delta - i\Gamma)$ is the atomic susceptibility operator, and $d_i$ are the atomic dipole operators. The subscript "$\perp$" denotes the reduced $2 \times 2$ operator, describing the $yz$ polarization plane [6]. We identify the retrieved light field as the eigenvector of $\left\langle \overleftrightarrow{\chi} \right\rangle_\perp$ with minimal imaginary eigenvalue, that is, the light field with minimal loss. When identifying the atomic quasi-dark state in the analysis of the storage process, we assumed time-invariant light fields $\Omega_\mathrm{c}$ and $\Omega_\mathrm{s}$. To identify the least decaying mode of the light, we correspondingly take a time-invariant (steady-state) atomic susceptibility $\left\langle \overleftrightarrow{\chi} \right\rangle_\perp$. Using the atomic state (4), the susceptibility tensor of the medium is given by

$$\langle \overleftrightarrow{\chi} \rangle_\perp = i\frac{d_{\mathrm{Cs}}^2}{\Gamma}\begin{pmatrix} 1-2ib_{\mathrm{Cs}}^2\frac{\Gamma}{\Delta}\eta_{\mathrm{A}} & -i\eta_{\mathrm{A}}\left(e^{i\phi_{\mathrm{A}}} - i a_{\mathrm{Cs}} b_{\mathrm{Cs}}\frac{\Gamma}{\Delta}e^{-i\phi_{\mathrm{A}}}\right) \\ i\eta_{\mathrm{A}}\left(e^{-i\phi_{\mathrm{A}}} - ia_{\mathrm{Cs}}b_{\mathrm{Cs}}\frac{\Gamma}{\Delta}e^{i\phi_{\mathrm{A}}}\right) & \eta_{\mathrm{A}} - \frac{i}{2}a_{\mathrm{Cs}}^2\frac{\Gamma}{\Delta}\end{pmatrix}. \tag{8}$$

The least decaying eigenvector $\mathbf{E}^{\mathrm{out}}$ of this matrix is given to first order in $\eta_{\mathrm{A}}$ and in $\Gamma/\Delta$ by

$$\mathbf{E}^{\mathrm{out}} = \begin{pmatrix} i\eta_{\mathrm{A}}e^{-i2\alpha}\left(e^{i\phi_{\mathrm{A}}} - i\alpha e^{-i\phi_{\mathrm{A}}}\right) \\ 1 \end{pmatrix}. \tag{9}$$

The resulting light field is elliptically polarized. We can relate the polar and azimuthal angles on the Poincaré sphere to those of the Bloch sphere by

$$\eta_{\mathrm{L}}^{\mathrm{out}} = \eta_{\mathrm{A}}\sqrt{1-2\alpha\sin\left(2\phi_{\mathrm{A}}\right)} \tag{10}$$

and

$$\phi_{\mathrm{L}}^{\mathrm{out}} = \frac{\pi}{4} - 2\alpha + \arctan\left[\frac{1+\alpha}{1-\alpha}\tan\left(\phi_{\mathrm{A}} - \frac{\pi}{4}\right)\right]. \tag{11}$$

Once again, these are the mathematical equations of an ellipse with the semi-major and semi-minor axes $\eta_{\mathrm{L}}\left(1 \mp \alpha\right)$, now rotated by an angle $\frac{\pi}{4}$. The mapping is exemplified graphically in supplementary figure 2 for $\eta_{\mathrm{A}} = 10^{-3}$ and $\alpha = 0.1$ with $0 \leq \phi_{\mathrm{A}} \leq 2\pi$. The ellipticity of the mapping, determined by the parameter $\alpha$, tilts the light polarization differently for different azimuthal phases.

Here again, in the ideal case $\alpha \to 0$, the medium becomes completely transparent to the state $\vec{E}^{\mathrm{out}}$, and supplementary equations (10) and (6) reduce to the simple linear relations $\eta_{\mathrm{L}}^{\mathrm{out}} = \eta_{\mathrm{A}}$ and $\phi_{\mathrm{L}}^{\mathrm{out}} = \phi_{\mathrm{A}}$.

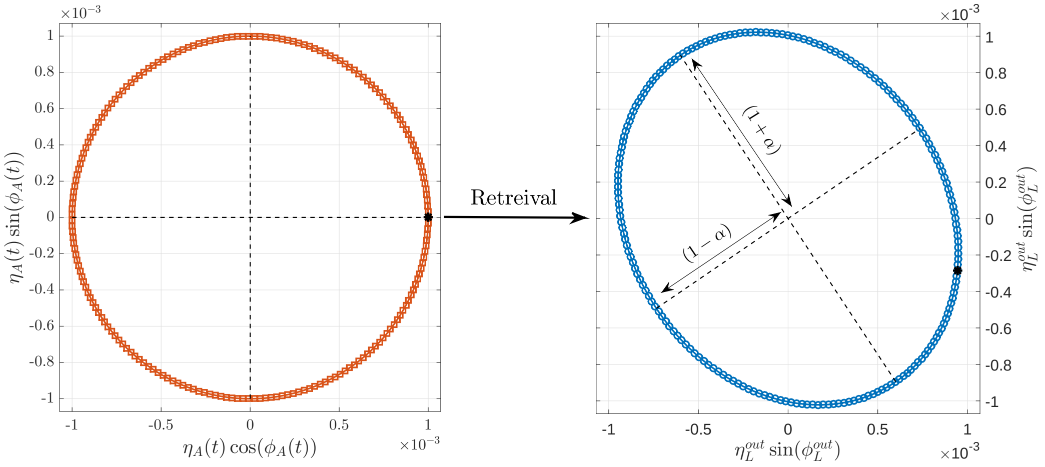


Supplementary figure 2: **Mapping of the atomic spin state to the light state at retrieval.** The transformation of the atomic state represented on the Bloch sphere to the photonic output state represented on the Poincare sphere is shown for$\eta_{\mathrm{A}} = 10^{-3}$, $\alpha = 0.1$. We mark the points $\phi_{\mathrm{A}} = 0$ and $\phi_L\left(\phi_{\mathrm{A}} = 0\right)$ (black asterisk) to illustrate the mapping of the azimuthal phase, which acquires a mean additional phase shift of $-2\alpha$.

## Supplementary note 3. Protocol for eliminating the ellipticity of the overall storage-to-retrieval transformation

The overall transformation of the storage, followed by a storage time $t$, and then retrieval, is described by

$$\eta_{\mathrm{L}} \to \eta_{\mathrm{A}}(0) \to \eta_{\mathrm{A}}(t) \to \eta_{\mathrm{L}}^{\mathrm{out}}, \tag{12}$$

$$\phi_{\mathrm{L}} \to \phi_{\mathrm{A}}(0) \to \phi_{\mathrm{A}}(t) \to \phi_{\mathrm{L}}^{\mathrm{out}}. \tag{13}$$

The first and third steps are given by supplementary equations (5), (6), (10), and (11). The second step describes the dynamics of the atomic spins in the dark for a duration $t$, governed by a Larmor precession,

$$\phi_{\mathrm{A}}(t) \quad = \quad \phi_{\mathrm{A}}(0) + \omega_B t, \tag{14}$$

and by decay due to the various relaxation mechanisms,

$$\eta_{\mathrm{A}}(t) = \eta_{\mathrm{A}}(0) e^{-t/\tau_{\mathrm{s}}}.$$

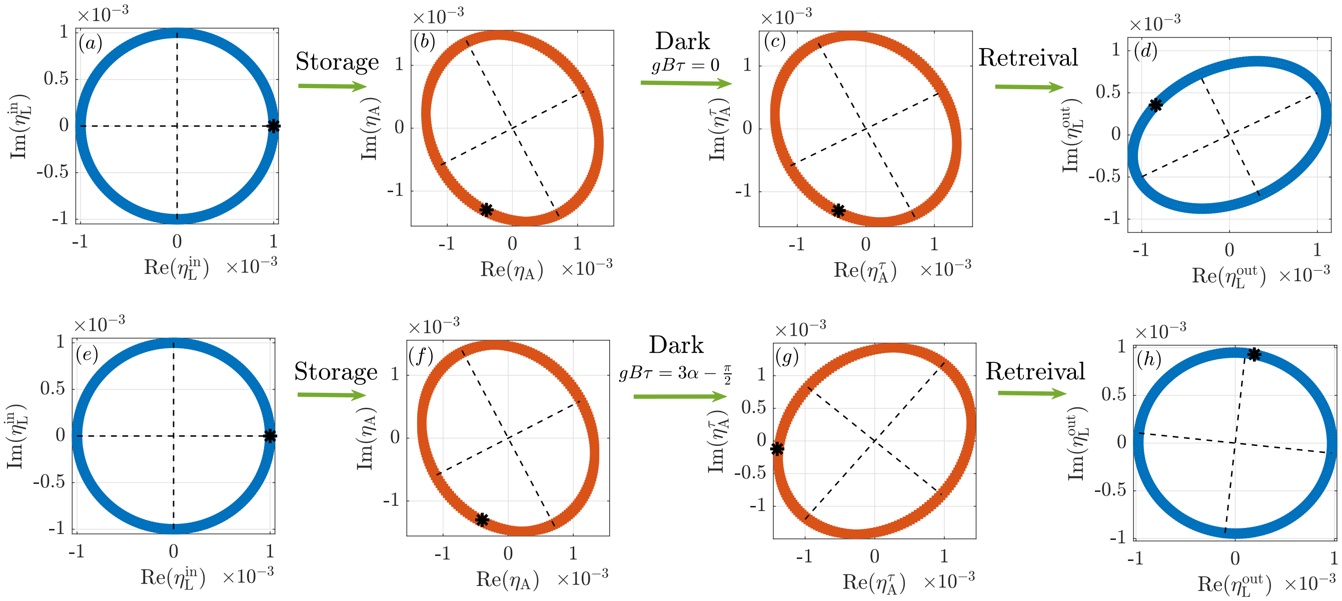


Supplementary figure 3: **Full transformation from storage to retrieval.** The transformation of the photonic input state to the photonic output state through storage and retrieval on the atomic state is shown for $\eta_{\mathrm{L}} = 10^{-3}$ and $\alpha = 0.1$. (a)-(d): For a vanishing Larmor precession $\omega_B t = 0$, the transformation is elliptical. (e)-(h): For $\omega_B t = 3\alpha - \pi/2$, the elliptical distortion is eliminated.

Ideally $\alpha = f_{\mathrm{Cs}}\Gamma/\Delta \ll 1$ and $t \ll \tau_{\mathrm{s}}$, such that supplementary equations (10) and (11) are the inverse transformation of supplementary equations (5) and (6), and the ground state relaxation is negligible, yielding perfect storage and retrieval of light. However, if $\alpha < 1$ but non-negligible (as in our experiment), then the retrieved signal is altered by the coupling to the off-resonant level $|p\rangle$ and given to first order in $\alpha$ by

$$\phi_{\mathrm{L}}^{\mathrm{out}} \approx \phi_{\mathrm{L}} + \omega_B t - 6\alpha - 2\alpha \cos\left(\omega_B t - 3\alpha\right) \cos\left(2\phi_{\mathrm{L}} + \omega_B t\right) \tag{15}$$

$$\eta_{\mathrm{L}}^{\mathrm{out}} \approx \eta_{\mathrm{L}} e^{-t/\tau_{\mathrm{s}}} \left(1 - 2\alpha \sin\left(2\phi_{\mathrm{L}} + \omega_B t\right) \cos\left(\omega_B t - 3\alpha\right)\right). \tag{16}$$

We see that, in general, the output light suffers the elliptical distortion twice, resulting in a storage efficiency that depends on the phase. This effect can be understood either as self rotation of the light polarization [7] or as arising from degenerate four-wave mixing [8].

The elliptical distortion can be removed completely by setting the magnetic field to satisfy $\omega_B t \approx 3\alpha - \pi/2$, see supplementary figure 3. For this value, the transformation simplifies to $\phi_{\mathrm{L}}^{\mathrm{out}} \approx \phi_{\mathrm{L}} - 6\alpha$ and $\eta_{\mathrm{L}} = e^{-t/\tau_{\mathrm{s}}}\eta_{\mathrm{L}}$, such that the amplitude $\eta_{\mathrm{L}}$ is independent of $\phi_{\mathrm{L}}$. We emphasize that the distortion is eliminated for all orders of $\alpha$ and is thus completely removed. The process can be described in terms of a perfect quantum eraser, since it neither depends on the (intermediate) spin states nor it involves any classical feedback or measurement of the quantum system.

## Supplementary References


[1] Fleischhauer, M., Imamoglu, A. & Marangos, J. P., Electromagnetically induced transparency: Optics in coherent media. Rev. Mod. Phys. 77, 633–673 (2005).

[2] Steck, D. A. http://steck.us/alkalidata (2009).

[3] Novikova, I., et al. Optimal Control of Light Pulse Storage and Retrieval. Phys. Rev. Lett., 98, 243602 (2007).

[4] Nunn J.,et al. Multimode Memories in Atomic Ensembles. Phys. Rev. Lett. 101, 260502 (2008).

[5] Happer W., Jau Y. Y. & Walker T. Optically pumped atoms. 159–218 (WILEY-VCH Press, 2010).

[6] B. S. Mathur, H. Y. Tang, and W. Happer. Light Propagation in Optically Pumped Alkali Vapors. Phys. Rev. A 2, 648 (1970).

[7] Novikova, I., Phillips, D. F. & Walsworth., R. L. Slow light with integrated gain and large pulse delay. Phys. Rev. Lett. 99, 173604 (2007).

[8] Xu, X., Shen, S. & Xiao, Y. Tuning the phase sensitivity of a double-lambda system with a static magnetic field. Opt. Exp. 21, 11705 (2013).
